# Notes on the genus *Episcaphium* Lewis (Coleoptera, Staphylinidae, Scaphidiinae) with description of a new species from China

**DOI:** 10.3897/zookeys.595.8784

**Published:** 2016-06-02

**Authors:** Liang Tang, Yue-Ye Tu, Li-Zhen Li

**Affiliations:** 1 Department of Biology, Shanghai Normal University, 100 Guilin Road, 1st Educational Building 323 Room, Shanghai, 200234 P. R. China

**Keywords:** Coleoptera, Staphylinidae, Episcaphium, new species, China

## Abstract

A new *Episcaphium* species collected from Yunnan Province of China is described as *Episcaphium zhuxiaoyui* **sp. n.**, and its diagnostic characters are illustrated. A new province record of *Episcaphium haematoides* is reported. A key to the *Episcaphium* species recorded from China is provided.

## Introduction

*Episcaphium* Lewis, 1893 is a small Asian genus of Scaphidiinae. Up to the present, eleven species of the genus have been known from the world, and six species have been known from China: *Episcaphium catenatum* Löbl, 1999 and *Episcaphium watanabei* Löbl, 2002 from Sichuan, *Episcaphium strenuum* Löbl, 1999 and *Episcaphium haematoides* Löbl, 1999 from Yunnan, *Episcaphium changchini* Sheng & Gu, 2009 from Shaanxi, and *Episcaphium dabashanum* Sheng & Gu, 2009 from Chongqing.

Recently, we examined some specimens of the genus, among them a new species and a new province record.

## Material and methods

Specimens were mainly collected by hand from decayed wood and fungi in broad-leaved forests and killed with ethyl acetate. For examination of the male genitalia, the last two abdominal segments were detached from the body after softening the specimens in hot water. The aedeagi were mounted in Euparal (Chroma Gesellschaft Schmidt, Koengen, Germany) on plastic slides. Photos of the aedeagi were taken with a Canon G9 camera attached to an Olympus SZX 16 stereoscope; habitus photos were taken with a Canon macro photo lens MP-E 65 mm attached to a Canon EOS 7D camera and stacked with Zerene Stacker (http://www.zerenesystems.com/cms/stacker).

The type specimens treated in this study are deposited in the following public and private collections:

NMPC
National Museum, Praha, Prague, Czech Republic 


SHNU
 Department of Biology, Shanghai Normal University, P. R. China 


## Taxonomy

### Episcaphium zhuxiaoyui sp. n.

Taxon classificationAnimaliaColeopteraStaphylinidae

http://zoobank.org/1DE9B6EA-C85E-4506-8123-BB6F3CDE9DA4

**Figures 1–4.**
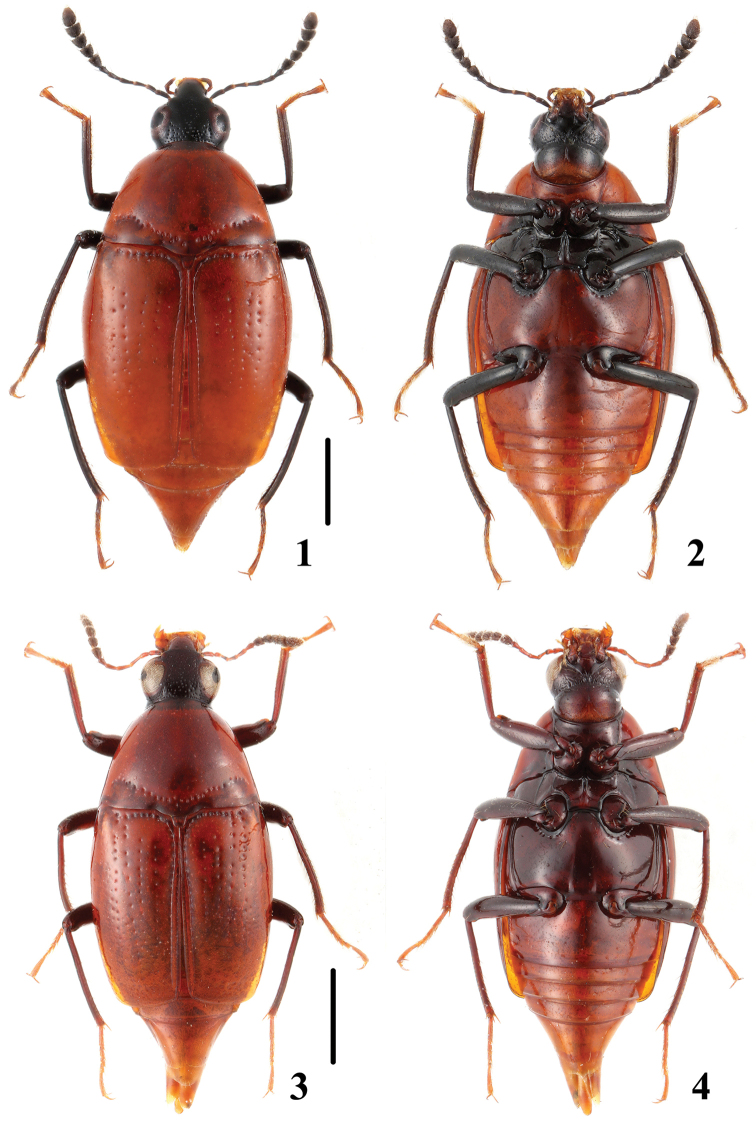
Adult habitus of *Episcaphium*. **1, 2** *Episcaphium zhuxiaoyui* sp. n. **3, 4** *Episcaphium semirufum* Lewis. Scales = 1 mm.

**Figures 5–10.**
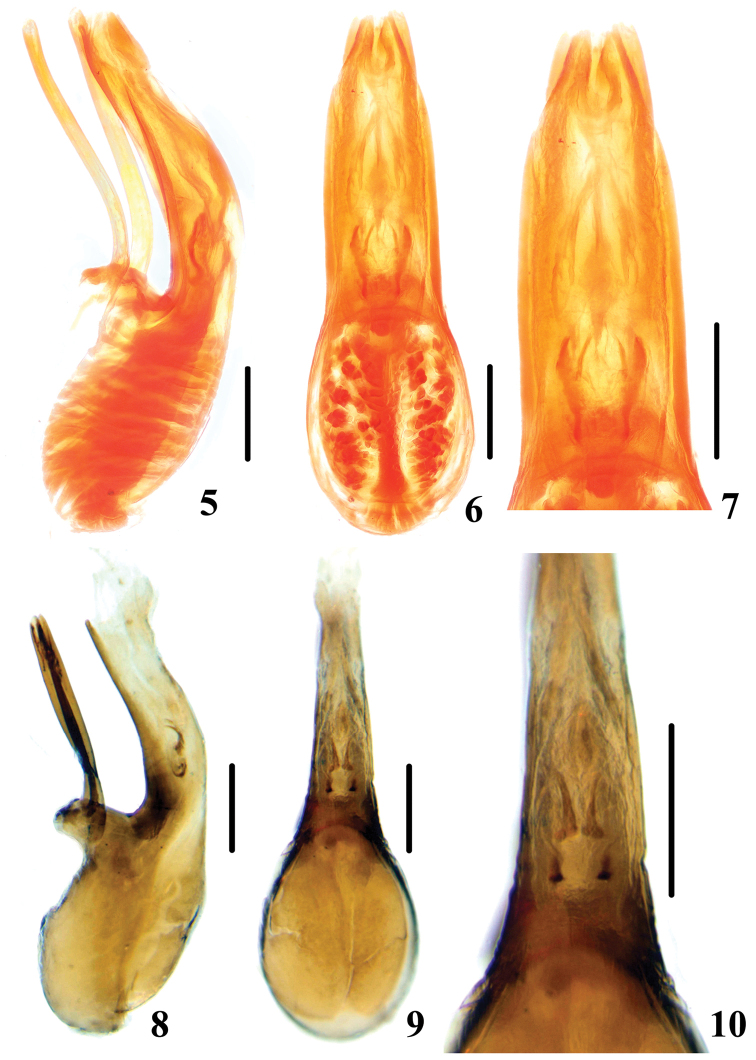
**5–7** *Episcaphium zhuxiaoyui* sp. n. **5** aedeagus in lateral view **6** aedeagus in dorsal view **7** details of internal sac **8–10** *Episcaphium semirufum* Lewis **8** aedeagus in lateral view **9** aedeagus in dorsal view **10** details of internal sac. Scales = 0.25 mm.

**Figures 11, 12.**
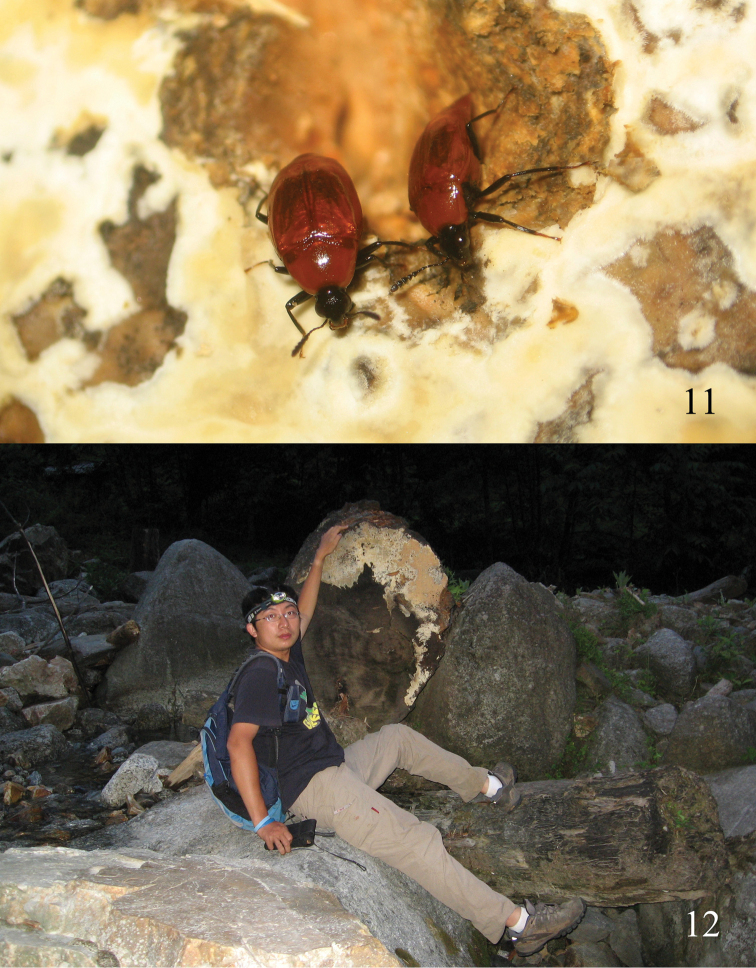
**11** Living *Episcaphium zhuxiaoyui* sp. n. on fungus **12** Collector Mr. Zhu Xiao-Yu and log with fungus. Photo by Mr. Jian-Qing Zhu.

Figs 1 , 2 , 5–7 , 11

#### Type material.

**Holotype. China: Yunnan**: ♂, glued on a card with labels as follows: “China, Yunnan Prov., Gongshan County, Heiwadi, alt. 2000 m, 7–10 June 2009, Zhu Jian-Qing & Zhu Xiao-Yu leg. ” “Holotype / *Episcaphium zhuxiaoyui* / Tang, Tu & Li” [red handwritten label] (SHNU) **Paratypes.** 5♂♂5♀♀, same data as the holotype (SHNU); 1♂, Deqin County, Nagu Vill., alt. 2250 m, 11.VII.2010, Wen-Xuan Bi leg. (SHNU); 7♂♂5♀♀, Lushui County, Laowo, Fenshuiling, alt. 2250 m, 7.VII.2010, Wen-Xuan Bi leg. (SHNU)

#### Description.

Body length: 5.3–5.9 mm. Pronotum width: 2.0–2.1 mm.

Head black, except for the reddish mouthparts. Inner basal parts of prohypomera, legs including coxal cavity and mesosternum blackish. Other parts reddish.

Frons at narrowest point 0.42–0.44 mm wide. Head coarsely and very densely punctate, punctation coarse on vertex and fine near eyes. Intervals between punctures distinctly smaller than diameter of punctures. Between eyes with a pair of impunctate patches. Labium smooth. Gular striae impressed, groove-like basally.

Pronotum with antebasal puncture row usually interrupted at middle (rarely uninterrupted), impressed laterally. Discal punctures fine and sparse.

Elytra with shallow apical impression and indistinct humeral protuberance; disc with four discal puncture rows consisting of rather coarse punctures anteriorly gradually becoming finer posteriad. All rows start at about basal 2/11 of elytron and end blurrily where puncture rows mix with apical disc punctures. Punctation fine between discal series of punctures and coarse in apical impressions. Mesoventral process with raised, ridge-like edges, and impressed in middle.

Metaventrite finely and sparsely punctate, lacking microsculpture, with medio-apical impression shallow, narrowed anteriorly, and carinate laterally.

Punctation of abdominal sternites very fine and very sparse. Micropunctures absent.

Male sexual characters. Segments 1 to 3 of protarsi slightly widened with dense setae on ventral side. Aedeagus (Figs 5, 6) with median lobe with apical portion inflexed in lateral view. Basal process small, slightly prominent. Parameres slightly sinuate in lateral view. Internal sac (Fig. 7) with a pair of comma-like sclerotized rods.

#### Distribution.

China (Yunnan).

#### Remarks.

This new species is similar to the variety of *Episcaphium semirufum* Lewis, 1893 with dark head described from Japan (Figs 3, 4, 8–10), but it may be distinguished from the latter by the different coloration of the ventral side, a pronotum with the antebasal puncture row usually interrupted at middle, and the formation of the discal puncture rows on the elytra: the two outer rows are distinctly separated from the basal puncture row, while in *Episcaphium semirufum*, they fuse with the basal puncture row. The new species is distinguished from all the other species by its coloration.

#### Etymology.

This species is named in honor of Mr. Xiao-Yu Zhu who collected some specimens of the new species.

#### Biological notes.

This species was found gathering on an unknown fungus on a huge rotten log across stream, and was observed to become active when night fell (Figs 11, 12).

### Episcaphium haematoides

Taxon classificationAnimaliaColeopteraStaphylinidae

Löbl, 1999

#### Material examined.

**China: Gansu**: 1♂, Lazikou Valley, 2020–2510 m, 34°09.9–10.1'N, 103°48.2–51.9'E, 28.VI.2005, J. Hájek, D. Král & J. Růžička leg. (NMPC)

#### Distribution.

This species was previously known from Yunnan and Sichuan. The above male represents the first record from Gansu.

### Key to *Episcaphium* species of China

**Table:** 

1	Pronotum black	**2**
–	Pronotum and elytra reddish, sometimes with black spots or fasciae	**4**
2	Elytra without puncture rows; abdomen reddish	***Episcaphium strenuum***
–	Elytra with four discal puncture rows; abdomen black	**3**
3	Pronotum with antebasal puncture row impressed laterally; elytra with distinct apical impressions	***Episcaphium dabashanum***
–	Pronotum with antebasal puncture row not impressed laterally; elytra without impressions	***Episcaphium catenatum***
4	Elytra reddish with black spots or fasciae	**5**
–	Elytra entirely reddish	***Episcaphium zhuxiaoyui***
5	Elytra each with two black transverse fasciae, without discal puncture rows	***Episcaphium watanabei***
–	Elytra each with one apical black spot, with four discal puncture rows	**6**
6	Tempora without punctures; pronotum with a pair of black basal spots situated between antebasal puncture row and basal edge	***Episcaphium haematoides***
–	Tempora densely punctate; pronotum with a pair of black median spots anterior to antebasal puncture row	***Episcaphium changchini***

## Supplementary Material

XML Treatment for Episcaphium zhuxiaoyui

XML Treatment for Episcaphium haematoides
